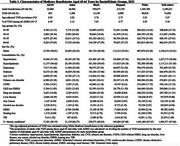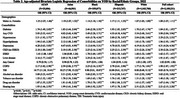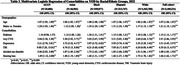# Young‐Onset Dementia in Medicare Beneficiaries: Prevalence and Comorbidities by Race and Ethnicity

**DOI:** 10.1002/alz70860_100620

**Published:** 2025-12-23

**Authors:** Jiahui Dai, Toubby Chau, María M. M. Corrada, Spero Manson, Joan O'Connell, Luohua Jiang

**Affiliations:** ^1^ Joe C. Wen School of Population & Public Health, Henry and Susan Samueli College of Health Sciences, University of California, Irvine, Irvine, CA, USA; ^2^ Northwestern University, Evanston, IL, USA; ^3^ University of California, Irvine, Irvine, CA, USA; ^4^ University of Colorado Anschutz Medical Campus, Aurora, CO, USA; ^5^ University of California Irvine, Irvine, CA, USA

## Abstract

**Background:**

Young‐onset dementia (YOD), which develops before age 65, can bring additional challenges to patients and their caregivers. The prevalence of YOD and its associated comorbidities across U.S. racial/ethnic populations remain unclear. Thus, this study aimed to estimate the prevalence of YOD and examine associations between past and current comorbidities (called comorbidities hereafter) and YOD among Medicare beneficiaries in various racial/ethnic groups.

**Method:**

This cross‐sectional study included Medicare beneficiaries aged 45‐64 years with almost‐continuous fee‐for‐service coverage in 2022. Data were extracted from the Center for Medicare and Medicaid Services 2022 Medicare Beneficiary Summary File (MBSF). MBSF race/ethnicity data were used to identify non‐Hispanic White (White), non‐Hispanic Black (Black), Hispanic, non‐Hispanic Asian (Asian), and non‐Hispanic American Indian and Alaska Native (AI/AN) populations. Comorbidities examined include diabetes, cardiovascular disease (CVD), hyperlipidemia, hypertension, depression, chronic kidney disease without end‐stage renal disease (ESRD), ESRD, liver disease, cancer, chronic obstructive pulmonary disease, traumatic brain injury (TBI), alcohol use disorder, drug use disorder, tobacco use disorder, and hearing loss. The outcome was YOD, defined as Alzheimer's disease and related dementias (ADRD) occurring in individuals aged 45‐64. YOD was identified using end‐of‐year indicators of ADRD from the MBSF 30 Chronic Conditions Data Warehouse.

**Result:**

In 2022, among a total of 2,189,231 Medicare beneficiaries aged 45‐64 years, 71,092 (3.27%) adults were diagnosed with YOD. Black adults had the highest age‐adjusted YOD prevalence (3.76%). Among beneficiaries aged 45 years and older with ADRD, the proportions of YOD were approximately two to three times higher among Black (7.01%), AI/AN (6.49%), and Hispanic (4.77%) individuals compared to White (2.89%) and Asian (2.12%) individuals (Table 1). Additionally, Black, Hispanic, and AI/AN adults had a higher prevalence of most examined comorbidities than White adults. Many comorbidities, such as TBI and CVD, demonstrated strong association with YOD (Table 2&3).

**Conclusion:**

Medicare beneficiaries under age 65 experienced a higher burden of YOD among racial/ethnic minority groups. Timely and accurate diagnosis of YOD, combined with enhanced health care coordination services, is critically needed for younger Medicare beneficiaries with YOD and multiple comorbidities.